# Supplementary figures and images for: Combined microbiome and metabolomics analysis of Taorong-type baijiu high-temperature Daqu and medium-temperature Daqu
Source: PeerJ. 2024 Jan 3;12:e16621. doi: 10.7717/peerj.16621 (PMC10771096; doi:10.7717/peerj.16621)

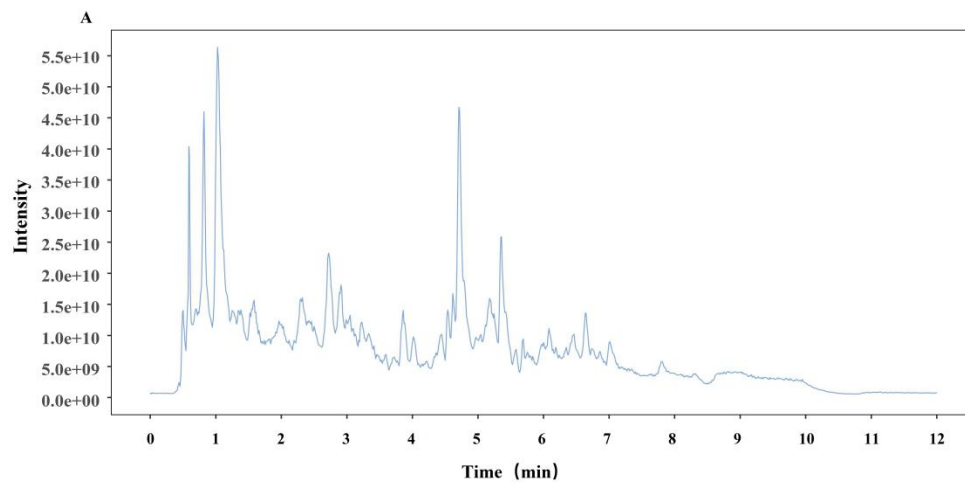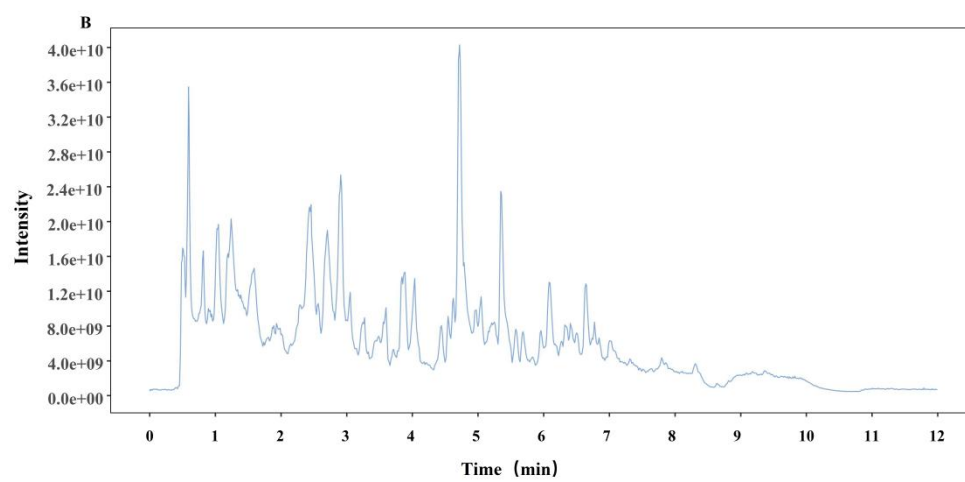

Supplement: Supplemental Information 1 [file peerj-12-16621-s001.pdf]
